# COVID-19 and Psychosocial Well-Being: Did COVID-19 Worsen U.S. Frontline Healthcare Workers’ Burnout, Anxiety, and Depression?

**DOI:** 10.3390/ijerph20054414

**Published:** 2023-03-01

**Authors:** M. Lelinneth B. Novilla, Victor B. A. Moxley, Carl L. Hanson, Alisha H. Redelfs, Jeffrey Glenn, Paola G. Donoso Naranjo, Jenna M. S. Smith, Lynneth Kirsten B. Novilla, Sarah Stone, Rachel Lafitaga

**Affiliations:** 1 Department of Public Health, Brigham Young University, Provo, UT 84602, USA; 2 J. Reuben Clark Law School, Brigham Young University, Provo, UT 84602, USA

**Keywords:** COVID-19, healthcare workers, burnout, anxiety, depression, resilience, self-efficacy

## Abstract

Healthcare workers are highly regarded for their compassion, dedication, and composure. However, COVID-19 created unprecedented demands that rendered healthcare workers vulnerable to increased burnout, anxiety, and depression. This cross-sectional study assessed the psychosocial impact of COVID-19 on U.S. healthcare frontliners using a 38-item online survey administered by Reaction Data between September and December 2020. The survey included five validated scales to assess self-reported burnout (Maslach Summative Burnout Scale), anxiety (GAD-7), depression (PHQ-2), resilience (Brief Resilience Coping Scale), and self-efficacy (New Self-Efficacy Scale-8). We used regression to assess the relationships between demographic variables and the psychosocial scales index scores and found that COVID-19 amplified preexisting burnout (54.8%), anxiety (138.5%), and depression (166.7%), and reduced resilience (5.70%) and self-efficacy (6.5%) among 557 respondents (52.6% male, 47.5% female). High patient volume, extended work hours, staff shortages, and lack of personal protective equipment (PPE) and resources fueled burnout, anxiety, and depression. Respondents were anxious about the indefinite duration of the pandemic/uncertain return to normal (54.8%), were anxious of infecting family (48.3%), and felt conflicted about protecting themselves versus fulfilling their duty to patients (44.3%). Respondents derived strength from their capacity to perform well in tough times (74.15%), emotional support from family/friends (67.2%), and time off work (62.8%). Strategies to promote emotional well-being and job satisfaction can focus on multilevel resilience, safety, and social connectedness.

## 1. Introduction

The United States (U.S.) has 22 million healthcare workers, representing 14% of the total national workforce [1]. Of these healthcare workers, 9.8 million are physicians, nurses, and technicians [1]. These workers are known for their excellence in the life sciences and for their patient skills, intense focus, and admirable dedication, often placing the well-being of their patients before their own. Society respects, as well as expects, that healthcare workers will be able to maintain their composure when faced with life-saving decisions and responsibilities.

In 2020, the COVID-19 pandemic plunged the entire world into an unfathomable individual and collective state of stress and fear. Worldwide, COVID-19 cases reached 83,832,334, with 1,824,590 deaths in 2020 [2]. In the U.S., more than 20 million cases and 346,000 deaths were reported by the end of 2020 [2]. By the second year of the pandemic, COVID-19 deaths in the U.S. surpassed the number of deaths from the 1918 H1N1 flu pandemic [3,4,5]. As the SARS-CoV-2 virus continued to evolve into highly transmissible variants, additional surges followed, especially as Omicron and its highly transmissible subvariants overtook the Delta strain [6].

The COVID-19 crisis created extraordinary challenges and demands that further strained an already-overstretched healthcare system. U.S. healthcare workers were thrust onto the frontlines in the fight against COVID-19, rendering them vulnerable to burnout, anxiety, and depression [7,8]. With a universally susceptible population, COVID-19’s first year was marked by pandemic exigencies that extended work hours, created social isolation, and made work–life balance far more difficult for healthcare workers [2]. Persistent surges in multiple hotspots across the country escalated patient volume, which overran hospital capacity in several locations [2,9]. Further, the virus’ rapid and unrelenting onslaught, particularly among seniors and at-risk populations, accelerated the demand for medical staff, personal protective equipment (PPE) [2,8,9,10,11,12], and ventilators while heightening the risk of exposure among frontliners and the transmission of infection to families and friends [12,13]. During the first six months of the pandemic, U.S. cities lacked testing supplies [14], a challenge compounded by the absence of definitive prophylactic and therapeutic drugs [15]. Social and emotional support through physical interactions with family and friends [16] was trumped by infectious disease protocols, particularly in healthcare settings, to control transmission. Understanding the potential vulnerability of frontliners given these unique COVID-19-related demands, the current study sought to explore COVID-19′s psychosocial impact on healthcare workers through measures of burnout, anxiety, depression, resilience, and self-efficacy.

### 1.1. Burnout

Burnout is the response to prolonged work-related emotional, physical, and mental stress that is detrimental to professional performance and social functioning [17,18]. While stress is primarily a nonspecific response to an environmental stimulus [19], burnout is the combination of a person’s outward reaction to others (depersonalization) and the inward reaction to self (decreased self-efficacy) as precipitated by emotional fatigue [17,18]. The 11th Revision of the International Classification of Diseases (ICD-11) emphasizes that rather than a medical condition, burnout is an occupation-related syndrome that has not been successfully managed and is characterized by energy depletion or exhaustion, negativism, and reduced professional efficacy [20]. Burnout is defined by three empirically derived dimensions: emotional exhaustion, depersonalization, and reduced personal accomplishment [17]. These dimensions are thought to arise sequentially; that is, an increased level of emotional exhaustion elicits a depersonalization response, which in turn results in feelings of inadequacy and helplessness [17,18,21].

Healthcare workers are prone to burnout and distress that can negatively impact work performance and quality. This can result in staff turnover and shortages and a higher risk of major medical errors and malpractice lawsuits that cost close to USD 5 billion per year in the United States [22]. A survey of U.S. healthcare workers showed that physicians are twice as likely to commit medical errors if they are experiencing both burnout and fatigue, while nurse burnout can lead to poor bedside manner, problems with patient relationships, and lack of work satisfaction [22]. During the first few months of the COVID-19 pandemic, 39% of healthcare workers, particularly nurses, reported feeling inadequately supported [22,23]. Further, burnout and distress can put clinicians at risk of suicide [22]. 

### 1.2. Stress, Anxiety, and Depression

Healthcare workers are vulnerable to stress, anxiety, depression, substance use disorders, and suicidal behavior [23,24]. Healthcare work, by its very nature, is demanding, challenging, and complex. Its occupational hazards include long hours, unexpected shifts, and on-call work. Further, it can be physically, emotionally, and socially taxing considering the caregiving demands; exposure to suffering, death, infections, and hazardous substances; and the multiple interactions with patients, their families, and peers at work [24]. Stress, anxiety, and depression can manifest as a variety of feelings, such as irritation, anger, denial, uncertainty, nervousness, anxiety, helplessness/powerlessness, lack of motivation, exhaustion, overwhelm, burnout, sadness, and depression, and may be accompanied by trouble sleeping and concentrating [16].

Anxiety is a common psychological condition. It is defined as a feeling of apprehension, dread, or foreboding, whether in stressful situations or not, characterized by “cognitive, somatic, emotional, and behavioral components” [25]. Although anxiety can be protective in threatening situations, severe and chronic anxiety can lead to an anxiety disorder that can impair individual functioning [25,26].

Studies have explored the relationship between burnout and anxiety and depression. Both anxiety and depression have been linked to the emotional exhaustion component of burnout [27]. However, burnout differs from anxiety and depression in that it is context-specific as an occupationally distinct response to work-related stressors [17,18]. Nevertheless, burnout is considered a risk factor and a predictor for anxiety [28,29]. Although the clinical features of burnout and depression have been observed to overlap, especially in cases of low work satisfaction and severe burnout, experts still consider burnout and depression as two distinct constructs [30,31,32,33]. 

### 1.3. Self-Efficacy and Resilience

Self-efficacy, as defined by Bandura, refers to “beliefs in one’s capabilities to organize and execute the courses of action required to produce given attainments” [34]. Self-efficacy is associated more with the belief that an individual can accomplish what they have set out to do rather than with their skills [35]. For this reason, self-efficacy is a better predictor of success than possessing actual skills, knowledge, or prior accomplishments [36,37]. 

Resilience is a complex and dynamic construct that has been conceptualized in diverse ways: (1) as an attribute, (2) as an outcome, and (3) as a process or mechanism. Despite the absence of a standard definition of resilience, researchers agree on its two-dimensional composition: significant adversity and positive adaptation [38,39]. As an attribute, resilience is seen as a personal trait or an individual quality of successfully bouncing back after an adverse experience [38]. This concept is reflected in earlier studies through the description of the “invulnerable child” [39]. Although the understanding of resilience grew out of this risk paradigm, subsequent waves of inquiry broadened the conceptualization of resilience to an outcome and a process promoted by factors external to the individual [40,41]. As an outcome, resilience results from positive adaptation despite experiences of significant adversity or trauma, in which positive adaptation represents the best possible outcomes within the context of risk [40]. As a process, resilience develops from adaptive mechanisms by which person–environment interactions permit promotive factors to positively modify the perception of risks in adverse life circumstances [40,42,43,44,45,46,47,48]. Masten’s definition of resilience emphasizes not only this adaptive process, but also the role of multiple interacting systems—individual, family, community, social network, and society—in the development of resilience [49].

### 1.4. Purpose of the Study

The objective of this study was to assess the psychosocial impact of COVID-19 on U.S. frontline healthcare workers by exploring the following questions: (1) What was the relationship between working in facilities located in COVID-19 epidemiological hotspots and the indicators of psychosocial well-being (burnout, anxiety, depression, resilience, and self-efficacy) among COVID-19 healthcare frontliners? (2) What were the changes in self-reported psychosocial well-being (burnout, anxiety, depression, resilience, and self-efficacy) before and during COVID-19? (3) What individual- and organizational-level risk factors influenced burnout before and during COVID-19 among COVID-19 healthcare frontliners? (4) Which resilience factors (assets and resources) were perceived to be protective by frontline healthcare workers during the COVID-19 pandemic?

A deeper understanding of the root causes of burnout, anxiety, and depression among healthcare workers is critical to patient care and safety, healthcare-worker retention, and the strength of the healthcare system. Currently, there is a lack of an interdisciplinary framework for addressing risks and protective factors while also nurturing individual, organizational, and system-level resilience. We anticipate that the results of this study can inform efforts in addressing the psychosocial well-being of healthcare workers, particularly those at the frontlines in the fight against COVID-19, to strengthen their capacity to adapt to both acute and prolonged occupational stress.

## 2. Materials and Methods

### 2.1. Study Design 

We conducted a cross-sectional study of frontline healthcare workers to assess the psychosocial impact of providing care during the COVID-19 pandemic. Reaction Data, a healthcare market research firm in Utah, helped administer an anonymous 38-item online survey to healthcare frontliners from September 2020 to December 2020. Reaction Data’s proprietary Research Cloud database consists of 800,000+ U.S. healthcare professionals, such as physicians, physician assistants, nurses, and others, from hospitals, clinics, and pharmaceutical companies [50]. Our study was approved by the Brigham Young University Institutional Review Board, and all survey participants read and signed an informed consent prior to participation.

### 2.2. Survey Instrument and Administration 

The survey instrument (Supplementary Materials, Table S1) examined the demographic characteristics of survey respondents (age, race/ethnicity, zip code, professional training, specialty area, seniority, and years in practice) and measured selected psychosocial variables and outcomes. The dependent or psychosocial variables included the following: (1) burnout (Maslach 2-Question Summative Scale, M2QSS) [51], (2) anxiety (Generalized Anxiety Disorder-7; GAD-7) [52,53], (3) depression (Patient Health Questionnaire-2 based on DSM-IV criteria; PHQ-2) [54,55], (4) resilience (Brief Resilience Coping Scale, BRCS) [56], and (5) self-efficacy (New General Self-Efficacy Scale-8, NGSES-8) [57]. We chose these scales because they are validated, reliable measures used in multiple scientific studies; they are based on the existing literature about the impact of COVID-19 on the mental health of healthcare workers; and they contain fewer survey questions than alternative options, making them suitable for the study’s target respondents, who have busy schedules. All scales showed adequate to good reliability in our sample, with Cronbach’s values ranging from 0.73 to 0.93 (Table 1). The scales included items that asked respondents about their current (during COVID-19) and prepandemic (before COVID-19) psychosocial conditions. Questions related to perceptions before COVID-19 were treated as within-individual controls by comparing respondents’ answers based on their current circumstances.

The independent variables included (1) risk and (2) resilience. Respondents were asked to identify which items from a list of risk and resilience factors applied to them. Risk factors included items such as fear of family or oneself becoming infected, uncertainty about the duration of the pandemic, and exhaustion from long working hours. Resilience factors included achieving goals, confronting and accomplishing difficult tasks, overcoming challenges, and performing well even when conditions are tough. The survey included two open-ended questions: “What aspects of your work make you feel the most stressed while treating COVID-19 patients?” and “What external factors and resources listed below (e.g., goals, family, community) help you cope with the stress of treating COVID-19 patients?”.

### 2.3. Recruitment 

In September 2020, Reaction Data sent out the first email survey invitation to a random sample of physicians, nurses, and other healthcare workers. The email included a cover letter with information about the study and the link to the online survey. Each potential respondent was given one week to complete the survey. Following a “cool-down” period, Reaction Data distributed a second email (October 2020) to target respondents who did not complete the survey following the first invitation.

### 2.4. Sample 

Our target respondents were randomly sampled from a sampling frame of frontline healthcare providers across the U.S. These included frontline healthcare professionals from a variety of medical and allied health specialties and subspecialties who, at the time of the survey, were working in several types of healthcare facilities and were directly caring for patients diagnosed with COVID-19. Responsibilities of target respondents included, but were not limited to, COVID-19 screening, hospital/clinic triage, laboratory diagnosis, radiology and imaging, respiratory therapy, treatment, nursing, rehabilitation, consults, referring, and management.

### 2.5. Power 

We conducted an a priori power analysis to calculate the sample size using the literature on psychosocial health among Chinese frontline healthcare workers in which cross-sectional samples ranged from 994 [58] to 2299 [59] subjects. Analysis revealed that a minimum of 267 subjects were needed to detect a difference in the proportion of clinicians experiencing burnout before and during COVID-19 [51] while accounting for resilience (alpha = 0.05, power = 80%).

### 2.6. Data Analysis

We conducted statistical analyses using SAS 9.4 and generated index scores for all psychosocial scales and descriptive statistics for all variables.

We coded respondents as working or not working in COVID-19 “hotspot counties” based on their county and publicly available COVID-19 incidence rates [60]. According to Oster, hotspot counties were defined as having met all 4 of the following criteria at least 1 day prior to the assessment date: (1) more than 100 new COVID-19 cases in the recent 7 days, (2) an increase in the most recent 7-day COVID-19 incidence compared with the preceding 7-day incidence, (3) less than 60% reduction in cases or an increase in the most recent 3-day COVID-19 incidence compared with the preceding 3-day incidence, and (4) 7-day incidence/30-day incidence ratio that exceeded 0.31. Hotspots must also have met at least 1 of the following criteria: (1) greater than 60% change in the most recent 3-day COVID-19 incidence or (2) greater than 60% change in the most recent 7-day incidence [60].

We used paired T-tests to compare scores for depression, anxiety, burnout, resilience, and self-efficacy during COVID-19 and before COVID-19. We evaluated between-group differences (T-tests) for the psychosocial variables comparing hotspot counties versus non-hotspot counties and by binary demographic variables such as gender. We assessed the differences in scores for categorical variables such as race/ethnicity (ANOVA) and the association of psychosocial index scores with age and tenure (Pearson correlation). We conducted a multiple regression analysis using stepwise automatic model selection (GLMSELECT procedure; criteria: overall model fit *p* < 0.05, added variable *p* < 0.05) to identify the relationships between demographics, stressors, and protective factors (resilience, self-efficacy) as independent variables and index scores for depression, anxiety, and burnout as dependent variables.

Analyzing open responses may be approached using either a deductive or an inductive approach [61]. The deductive approach allowed us to align our research questions with the preset literature-based categorical response options, giving us the advantage of maintaining our focus on the aims of our research. Survey Questions 24, 29, and 30 were multiple-response questions, in which more than one answer could be selected. Question 24, on the most stressful aspects of work, and Question 29, on external factors and resources for coping, offered preset options to choose from and also provided an opportunity to include a free response. On the other hand, Question 30, on individual or internal assets for coping, had 8 preset response options and did not include a free-response option. The preset response options for Questions 24, 29, and 30 were based on the existing literature on COVID-19 and its impact on healthcare workers [59,62,63,64,65]. These options represented the topics of interest covered by the study’s research questions, which we defined prior to the analysis as opposed to identifying them as they emerged while coding the data. First, we read all free responses (immersion in the data); second, we sorted the responses according to the preset literature-based categories, which form a thematic framework; third, we systematically applied the thematic framework to the data; and fourth, we added new themes as they emerged. The use of a deductive approach in analyzing the free responses allowed for a more structured analysis that drew from the existing knowledge on the topic. The lead author (MLBN) completed the qualitative analysis, which was verified by the corresponding author (VBAM).

## 3. Results

A total of 557 frontline healthcare workers responded to the survey; 289 were male (52.4%) and 262 were female (47.5%), with a median age of 56 years old (Table 2). Of the 557 respondents, 79.2% self-reported their race/ethnicity as Caucasian, 10.0% as Asian or Pacific Islander, 4.0% as Hispanic or Latino, and 3.1% as Black. The majority of respondents (87.2%) were practicing in hospitals or clinics located in urban areas, with two-thirds (63.6%) in counties categorized as COVID-19 hotspots. Almost half of the respondents (46.0%) were from the Eastern region of the U.S.

In terms of professional discipline, respondents were primarily physicians (85.4%) and nurses (12.8%) working in acute care hospitals (45.8%) or outpatient clinics/medical offices (40.3%). The most commonly reported medical specialties were internal medicine (21.6%) and pediatrics (21.0%). As for years of practice, a majority (59.4%) of the physicians had been practicing for 22 years or longer, 34.3% had been practicing for 10–19 years, and 6.0% had been practicing for 1–9 years. Further, 39.2% of respondents had management or executive leadership roles. When asked about the impact of COVID-19 on their practice, 99.5% of respondents reported that the pandemic had at least some impact on their practice, with 60.44% reporting a high-to-severe impact on their professional practice (see Table 3).

### 3.1. Epidemiological COVID-19 Hotspots and Psychosocial Impact (Burnout, Anxiety, Depression, Resilience, and Self-Efficacy) among COVID-19 Healthcare Frontliners

The survey was administered during the last quarter of 2020 (September–December 2020). This phase of the pandemic in the U.S. was marked by continued surges in cases and by the rising number of deaths from COVID-19. Because the classification of a healthcare facility as being located in a COVID-19 hotspot may have had an impact on psychosocial health given the increase in patient load, long work hours, and the demand for equipment and resources, we further explored this variable in the analysis. The results showed that there were no significant differences in burnout, anxiety, depression, resilience, or self-efficacy between healthcare workers in facilities located in epidemiological COVID-19 hotspots and healthcare workers in non-COVID-19 hotspots. The direct care of patients with COVID-19 also did not have a significant relationship with psychosocial well-being (Supplementary Materials, Table S2). 

### 3.2. Changes in Self-Reported Psychosocial Impact (Burnout, Anxiety, Depression, Resilience, and Self-Efficacy) before and during COVID-19 among COVID-19 Healthcare Frontliners

COVID-19 negatively affected the mental health of healthcare frontliners; amplified preexisting self-reported burnout, anxiety, and depression; and reduced resilience and self-efficacy (Table 4). Compared with prepandemic self-reported recall scores, burnout, anxiety, and depression (negative psychosocial conditions) increased significantly (54.8%, 138.5%, and 166.7%, respectively) during the first 8–9 months of the pandemic, while resilience and self-efficacy (positive psychosocial conditions) declined (6.5% and 5.7%, respectively). In terms of gender differences in reporting psychosocial conditions, female frontline healthcare workers were more likely to report burnout, anxiety, and depression compared with their male counterparts. However, this result was found to be not statistically significant in this study (Supplementary Materials, Table S3).

### 3.3. Individual- and Organizational-Level Risk Factors for Burnout before and during COVID-19

The scores on the burnout scale were predicted using stepwise multiple regression for both pre-COVID-19 and during COVID-19. The n for the pre-COVID-19 sample was 385 and the n for the during-COVID-19 sample was 357, with the remainder of the cases being dropped due to missing data on at least one variable. As a stepwise multiple regression was used, each independent variable was entered one at a time, as determined by SAS, until optimal fit was achieved (selection criterion of significance level, with entry set at 0.05). Results for these stepwise multiple regression analyses are summarized in Table 5 and Table 6. We also added the sr^2^ unique values [66] to Table 5 and Table 6 to provide information about the unique amount of variance in the burnout outcomes explained by each individual predictor variable in the models (i.e., after accounting for the variance explained by the other predictor variables in the models). In Table 5, age, assets/goals, and level of resilience pre-COVID-19 explained the most variance in burnout before the pandemic. On the other hand, in Table 6, the level of pre-COVID-19 resilience; exhaustion as a stressor; and age explained the most variance in burnout during the COVID-19 pandemic.

The overall pre-COVID-19 regression model, including all independent variables, was statistically significant with the pre-COVID-19 R = 0.366, R^2^ = 0.134, adj R^2^ = 0.122, F(5, 384) = 11.19, *p* < 0.001. However, only 13% of the variance in burnout scores pre-COVID-19 was accounted for by the variables included in the regression model. Among pre-COVID-19 healthcare frontliners, burnout was positively associated with psychological stress due to the lack of staff and resources and poor mental health, while age, achieving goals, and resilience were protective.

The “during-COVID” regression model was statistically significant: R = 0.508, R^2^ = 0.258, adj R^2^ = 0.245, F(6, 356) = 20.30, *p* < 0.001. All the individual variables were statistically significant as well. Burnout during COVID-19 could be predicted better than before COVID-19, with approximately 26% of the variance in burnout scores accounted for by the final regression model. Self-reported burnout before COVID-19 was significantly associated with an individual’s burnout during the pandemic, as were stressors such as exhaustion from working long hours and the shortage of resources (an organizational-level risk factor) such as medical staff, PPE, and equipment. However, age, achieving most of one’s personal goals, and resilience in overcoming challenges (individual-level factors/internal assets) were protective against burnout “during COVID” (Supplementary Materials, Table S4).

Both before and during the pandemic, achieving individual goals was most highly associated with reduced burnout. An increase of 1 unit in achieving personal goals was associated with a decrease of 0.79 to 0.81 on the burnout scale before and during the pandemic.

Based on our analysis of the free-response questions, the top three work stressors identified by respondents were uncertainty about the end of the pandemic and/or return to normal (54.8%); anxiety/fear of infecting family members, such as one’s child(ren), spouse, or older family member (48.3%); and internal conflict between protecting oneself and fulfilling the moral and ethical duty to care for patients (44.3%) (Table 7).

### 3.4. Resilience Factors (Individual Assets and Organizational Resources) Perceived to Be Protective during COVID-19 

Both individual and organizational factors were perceived by respondents to be protective during COVID-19 as they enhance coping. At the individual level, the top three personal resilience assets focused on self-efficacy, such as deriving strength from (1) belief in one’s capacity or personal bandwidth to perform well during tough times (74.2%), (2) confidence in one’s ability to effectively perform various tasks (55.8%), and (3) capacity to face and accomplish difficult tasks (52.2%). The top three external or organizational-level resilience resources included (1) “Other” (85.3%), a category comprised of a mix of resilience assets and resources at the individual level (faith, prayers, religious beliefs; exercise; rest/relaxation, nature walks, hobbies; personal improvement/personal study such as learning a new language; avoiding social media/news), family level (family/spouse support), community level (support from faith community), workplace/organizational level (support at work from patients and colleagues by “talking at work about challenges and opportunities”, debriefing with colleagues, having “supportive Zoom meetings”, “no COVID patients to take care of”, adhering to prevention and infection control measures, “appropriate resources for patient care”, “employer is sending emails thanking us for our contribution but cutting our salary by 70 percent”, “more transparency from admin and a bonus”), and at the government/societal level (avoiding election news, having “a systematic cohesive federal response to Covid-19 based on science”, and avoiding politicizing the pandemic); (2) receiving emotional support from family and friends (67.2%); and (3) having a flexible schedule and time off work (62.8%), which was closely followed by having a positive atmosphere at work (positive attitude of colleagues/peers) (Table 8). 

## 4. Discussion

The COVID-19 pandemic created greater awareness about the plight of healthcare workers. As a self-reported snapshot of psychosocial well-being, our study confirmed the negative impact of the extraordinary stress borne by medical frontliners during an unprecedented time in world history, a finding that has been demonstrated by other studies [62,63,64,65,67,68,69,70,71,72,73,74,75,76].

### 4.1. Changes in Self-Reported Psychosocial Conditions (Burnout, Anxiety, Depression, Resilience, and Self-Efficacy) before and during COVID-19 among Frontline Healthcare Workers

Even before the COVID-19 pandemic, healthcare workers faced high levels of burnout that can have serious ramifications on patient and worker safety, quality of care, patient satisfaction, worker retention, and healthcare costs. In our study, the perceived psychosocial well-being of U.S. frontline healthcare workers was worse during the pandemic than before the pandemic. COVID-19 amplified preexisting burnout (54.8% increase), anxiety (138.5% increase), and depression (166.7% increase), and reduced resilience (5.7% decrease) and self-efficacy (6.5% decrease) among 557 respondents (52.6% male, 47.5% female). These findings demonstrate that the pandemic and its associated stressors aggravated negative psychosocial conditions, such as burnout, anxiety, and depression, but lessened positive psychosocial conditions, such as self-efficacy and resilience, by possibly intensifying preexisting healthcare challenges while introducing new demands, challenges, and burdens that exacerbated workplace stress. This result is consistent with our hypothesis that COVID-19 negatively affects multiple psychosocial conditions, both negative and positive, among healthcare workers. Our finding also aligns with a scoping review of the literature that showed that COVID-19 was an independent mental health stressor that led to stress, anxiety, depression, and insomnia [62]. 

An unexpected finding in our study was the overall association between COVID-19 and psychosocial conditions regardless of whether healthcare workers were treating patients with COVID-19 at the time of the study. This result was likewise affirmed by the lack of significant correlation between selected psychosocial conditions and working in facilities located in COVID-19 hotspots. We also did not see a significant impact of medical specialty on depression as there were no changes in pre-COVID-19 and during-COVID-19 scores. These findings imply the potential influence of (1) cumulative or compounded stress collectively endured by healthcare workers at the time of the survey, including the intense societal stress during which healthcare workers had to perform their jobs; (2) potential exposure to COVID-19 (in COVID-19 patient rooms and/or working on the same floor where COVID-19 patients were admitted) [23]; and (3) multiple mechanisms through which COVID-19 and its related stressors, including increased patient workload, affected the well-being of healthcare workers.

The correlation between COVID-19 and negative psychosocial conditions in our study appeared to be mitigated by age, gender, years of practice, professional/administrative rank, work hours, and levels of self-efficacy and resilience [62]. Our results showed that older age, higher self-efficacy, and higher resilience were associated with lower rates of depression, while longer work hours were associated with increased depression, a finding seen in other studies [63,72]. Older age was associated with increased resilience [63] and less burnout, possibly due to greater experience and familiarity with handling workplace stressors [72]. 

The gender-based difference noted in this study on the reporting of negative psychosocial conditions, though not statistically significant, was found to be higher among female healthcare workers (pre-COVID-19: 39.1% female to 33.2% male; during COVID-19: 62.5% female to 45% male). This finding aligns with the results of other studies conducted during the pandemic in which female frontline healthcare workers, particularly nurses, were more likely to report negative mental health symptoms compared with their male counterparts [23,63,64,65,69,73,74,75]. This result may be explained by the differences in the self-rating of health status between males and females, with a general lower self-perceived health status among women; the heavier work responsibilities commonly borne by women; and the nature and scope of the overall roles and responsibilities of female healthcare workers that chronically expose them to multiple stressors at work and at home. In contrast to other studies that had a larger subset of women and/or female nurses and that found that being female was inversely correlated with depression [63,64,65,69], our sample had fewer female nurses. In addition, our study found that covariates, such as resilience and self-efficacy, were lower among female healthcare providers, which could also be explained by the multiple work and home stressors that female healthcare workers are typically subjected to. 

We also noted that longer duration of professional practice correlated with lower levels of burnout, anxiety, and depression. This finding is consistent with the results of the State of Well-Being Report that found that levels of workplace distress decreased with increasing years in practice, particularly more than 25 years [75]. It is possible that the more experienced healthcare workers and workers in administrative positions have more autonomy and flexibility with regard to work hours and responsibilities, which may insulate them from the stresses faced by younger healthcare workers. 

### 4.2. Individual- and Organizational-Level Risk Factors for Burnout, Anxiety, and Depression before and during COVID-19 among Frontline Healthcare Workers 

Conditions at work are considered to be far more predictive of burnout than personal factors [22]. The imbalance between individual factors and contextual factors within the work setting promotes burnout. Six factors have been identified as critical to the person–job fit: workload, “lack of control”, “insufficient reward”, “breakdown of community”, “absence of fairness”, and “value conflict” [23]. The greater the gap between job and employee factors, the higher the likelihood of burnout. 

Prior to the COVID-19 pandemic, U.S. physicians were already five times more likely to experience burnout compared with workers in other professions [25], with almost 44% reporting at least one burnout symptom [26]. Almost 67% of U.S. healthcare workers screened positive for burnout [27], while another study reported moderate-to-severe burnout among U.S. emergency department healthcare workers (nurses 51.3%, doctors 45.7%) [28]. Thus, research on burnout among healthcare workers, especially physicians, is not new to the research community. The increased attention given to healthcare-worker burnout may be explained by the correlation between burnout and higher medical errors and lower overall performance [77]. 

In our study, higher depression was reported among healthcare workers who worked long hours. Respondents identified that they experienced (1) worry over the undetermined duration of the pandemic (54.8%), (2) fear/anxiety about infecting family members (48.3%), and (3) internal conflict about protecting self as opposed to fulfilling the moral and ethical duty to care for patients (44.3%) as individual- and organizational-level risk factors for burnout, anxiety, and depression. The long work hours and depression may be explained by physical exhaustion, which in turn could have increased susceptibility to burnout, anxiety, and depression. Further, lower tolerance to the unpredictability of the pandemic and pandemic-related events could have lowered self-efficacy and resilience [22,23,68]. Thus, in this study, although healthcare frontliners believed that it was their moral and professional duty to work long hours during the pandemic, fatigue and “value conflict” may have reduced their confidence in their ability to perform well under extended work hours. What respondents outwardly expressed as fear of COVID-19 infection or transmission may have stemmed from the inner lack of control over their personal and family safety considering the persistent rise in COVID-19 deaths during the first year of the pandemic.

Our study validated the existence of mental health issues among healthcare workers who were at the frontlines in the fight against COVID-19. Our findings are congruent with the Mental Health America (MHA) survey that found that a majority (93%) of healthcare workers reported stress, with 86% reporting anxiety and 76% reporting both exhaustion and burnout [23]. Factors that increased the risk for negative psychosocial indicators among the MHA respondents were consistent with the findings of our study (except for parenting challenges), such as being “stressed and stretched too thin”, being “worried about exposing loved ones”, being “emotionally and physically exhausted”, “not getting enough emotional support”, and “struggling with parenting” [23]. A study by Lai et al. (2020) in Wuhan and Hunan, China, where the first cases of COVID-19 were identified, reported an increase in anxiety, depression, insomnia, and psychological stress among 1200 healthcare workers who were directly involved in the diagnosis, treatment, and care of more than 10,000 COVID-19-positive patients [63]. Lai et al.’s findings are consistent with the results of our study, including the higher likelihood of female healthcare frontliners reporting mental health symptoms [63]. What was not explored or noted in our study was the presence of somatic signs and symptoms, such as appetite changes, headache/stomachache, insomnia, and other sleep disturbances, associated with negative psychosocial conditions [23,73]. 

### 4.3. Individual Assets and External/Organizational-Level Resilience Resources Perceived to Be Protective by Frontline Healthcare Workers 

To date, most published studies on the psychosocial health of COVID-19 frontline healthcare professionals have focused on burnout, anxiety, and depression. However, the current literature does not equally address the capacity of frontline healthcare workers to positively respond to, adapt to, and recover from stress and adversity during the COVID-19 pandemic. 

Resilience is vital in healthcare. Higher levels of resilience were associated with decreased levels of stress, anxiety, fatigue, and sleep disturbances among healthcare workers, factors that could compromise performance and patient care [76]. Studies have investigated resilience using a socioecological framework, which highlights the interactions between vulnerability and promotive factors at various socioecological levels: individual, family, community, work, and government/society [39,48,49]. Vulnerability factors exacerbate the negative effects of adversity [40], while promotive factors neutralize such effects [29,40]. Promotive or resilience-promoting factors that are internal to the individual are referred to as assets, while factors that are external to the individual are referred to as resources [40,78]. The interactions between risk and promotive factors determine the level to which resilience is protective [40]. As such, individual and organizational factors can serve as resilience-promoting assets and resources. 

Our study showed that internal assets (achieving goals, accomplishing difficult tasks, overcoming challenges, and having the capacity to perform well during tough times) were associated with higher resilience scores in the final regression models, as were holding administrative or leadership positions. There was a positive correlation between age, internal assets, and self-efficacy pre-COVID-19, while work stressors (exhaustion from working long hours and shortages of staff, PPE, and equipment) reduced self-efficacy. In other words, healthcare workers who, even before the pandemic, had confidence in their ability to achieve and perform well during difficult circumstances, were in positions of responsibility in the workplace, and were older at the time of the survey were more likely to be resilient during the pandemic. Healthcare workers who had already been dealing with work fatigue prior to COVID-19 were less likely to have the confidence to handle stressors at work under more challenging circumstances [68].

At the individual level, the top three internal resilience assets in our study that were strongly linked with self-efficacy included (1) belief in having the personal bandwidth to perform well during tough times (74.2%), (2) confidence in the ability to effectively perform various tasks (55.8%), and (3) capacity to face and accomplish difficult tasks (52.2%). The top three external or organizational-level resilience resources were (1) “Other” (85.3%), a category comprising a mix of resilience assets and resources at various socioecological levels, (2) receiving emotional support from family and friends (67.2%), and (3) having a flexible schedule and time off work (62.8%), which was closely followed by having a positive atmosphere at work (positive attitude of colleagues/peers). Thus, our findings show the need for resilience factors at various levels (individual, family, community, work, and government/society), which can serve as positive coping mechanisms for countering COVID-19-related stressors in the workplace. Although not explored in our study, resilience was found by other researchers to be positively associated with physical health, gratitude, and a sense of fulfillment [76,79]. In our study, workplace emotional support was considered vital by healthcare workers, a finding similar to results reported by the MHA study [23]. Organizational resilience, through training in healthier forms of coping, contributes to individual resilience and was identified as essential to prepandemic preparation [80]. Experts have likewise articulated the need to support the mental health and resilience of medical frontliners as part of the global recovery from the COVID-19 pandemic [81]. 

Resilience scores in our study were lower during COVID-19 among respondents experiencing feelings of uncertainty about the length of the pandemic or the uncertain return to normal. Given the positive relationship between self-efficacy and resilience, it is possible that lower tolerance for uncertainty reduced belief in the ability to bounce back from adverse circumstances, hence the lower resilience scores [68]. Moreover, as Di Trani et al. (2021) and Di Monte et al. (2020) reported in their respective studies, there was a negative correlation between burnout and the ability to tolerate uncertainty. This finding was likewise accompanied by the increased desire for predictability, particularly among individuals who had a high-risk burnout profile [68,82].

Our study also found higher self-efficacy and resilience scores and less burnout with increasing age, longer years in practice, and greater work–life balance [83]. We observed a slight correlation between age and resilience (R = 0.3). It is conceivable that the more chronologically mature and more experienced subset of respondents had greater familiarity with various stresses that were either innate or related to the medical profession. Considering that most healthcare workers in our study were physicians with over 16 years of experience, length of practice and holding a senior administrative position may have had a protective effect, possibly through greater control over workplace conditions. Owing to the novelty of the experience with COVID-19 and its associated challenges, studies are still emerging on the resilience of U.S. healthcare workers during the pandemic and on the role of individual- and organizational-level assets and resources.

### 4.4. Limitations and Future Research Directions 

Our study has several limitations. First, because we used a cross-sectional study and our findings represent only a snapshot of the emotional well-being of respondents at the time of the survey, caution should be exercised in interpreting the results. Second, the survey was conducted during the last quarter of the first year of the pandemic (2020), which was characterized by evolving scientific data on SARS-CoV-2; persistent surges in COVID-19 cases; and scarcity of PPE and testing resources, including the lack of a definitive treatment regimen and vaccines. Thus, the results of this study apply to this specific phase of the pandemic, and more research is needed to confirm our findings. Third, we used self-reported burnout, anxiety, depression, self-efficacy, and resilience measures that may lend to participant recall bias. Recall bias is a limitation of surveys that require self-reporting or self-rating, which in the case of our study focused on the respondents’ perceived psychosocial health prior to COVID-19, which was about 6–9 months before they completed the survey. Such bias could have led respondents to assess their “pre-COVID-19” psychosocial condition to be better compared with “during COVID-19.” In this case, this would have led to a much higher increase in the difference between their “during COVID-19” and “pre-COVID-19” psychosocial health. Additionally, our study did not ask respondents about home-related stressors such as childcare, home schooling, personal finances, and work–family balance, which could likewise have influenced levels of burnout, anxiety, depression, self-efficacy, and resilience. 

The majority of respondents in this study were White, which precluded further exploring the experience of burnout, anxiety, and depression based on ethnicity. In addition, it is possible that medical frontliners dealing with greater burnout and lower resilience were less likely to complete a voluntary survey. On the other hand, frontliners that completed the survey may have represented the more resilient or less work-stressed portion of healthcare providers. This possibility could potentially inflate measures of reliance and self-efficacy while potentially suppressing measures of burnout, anxiety, and depression. Several of the variables of interest were collinear, thus concealing the contributions of other variables from the model selection. The values for each of the psychosocial scores, prior to and during COVID-19, were collected concurrently and relied on respondents’ recollection of their condition earlier in the year, which could have potentially biased the differences due to COVID-19. Although the scores for the psychosocial scale from our sample were slightly skewed, possibly from recall bias, we are confident in having used preexisting validated measures with previously demonstrated distributions, and regression, which is a robust statistical test. Most of the respondents in this study were older physicians, with a median age of 56 years old, who had been in practice for more than 16 years and occupied positions of leadership at the time of the survey. Thus, this sample of respondents possibly had more control of their time and could respond to a voluntary survey. Future studies on healthcare-worker psychosocial well-being can address these limitations.

## 5. Conclusions

Burnout, anxiety, depression, and the worsening of preexisting psychosocial conditions among healthcare workers are among COVID-19’s hidden costs. Pandemic-associated stressors exacerbated the inherently demanding nature of healthcare work. Long work hours; increased patient load; staff shortages; social isolation; the lack of PPE, equipment, and resources; and high transmission rates fueled psychosocial distress among healthcare workers, making it a parallel pandemic that demands the nation’s attention. The imbalance between individual-level resilience assets and workplace demands renders physicians, nurses, and other medical professionals vulnerable to burnout, anxiety, and depression while negatively affecting self-efficacy and resilience. 

The confluence and interactions of individual factors, workplace demands, and societal conditions in a pandemic setting are complex. Although anxiety and depression are individual-level conditions, burnout is a job-related response to stress. The roots of burnout can be traced to the workplace environment, culture, and organization. Thus, targeted strategies for promoting well-being and job satisfaction need to focus on multilevel resilience, safety, and social connectedness. 

The findings of this study support the need to identify both individual- and organizational-level risks and protective factors and to implement effective ways to nurture self-efficacy and resilience among healthcare professionals, particularly those on the COVID-19 frontlines. Most studies on burnout offer self-efficacy and resilience recommendations at the individual level. However, COVID-19 has shown that opportunities for creating a culture of well-being at the institutional level abound and are necessary in building a resilient workforce. It is important for the healthcare system to review workplace priorities, policies, and practices to determine how these can negatively impact psychosocial well-being, particularly during unusually challenging and uncertain circumstances. 

The psychosocial well-being of healthcare workers is vital to a well-functioning healthcare system and to the nation’s health. Supporting our healthcare workforce requires enacting upstream measures and establishing an evidence-based systemic framework for preventing and addressing healthcare-worker burnout, anxiety, and depression even before they begin their professional careers. In response to COVID-19’s unprecedented demands on the healthcare workforce, we need to listen to our healthcare workers to better understand the full brunt of COVID-19′s impact on their psychosocial well-being while simultaneously finding ways to enhance individual and collective resilience. As the world prepares for future pandemics and moves toward a new but meaningful normal, we need to learn from COVID-19′s enduring lessons and heed the call for supporting our healthcare workers’ health, safety, and well-being.

## Figures and Tables

**Table 1:** Internal consistency of validated psychosocial scales.

Psychosocial Scale	Pre-COVID-19	During COVID-19
	Cronbach’s a (n)	Cronbach’s a (n)
M2QSS: Burnout [51]	0.73 (546)	0.73 (509)
GAD-7: Anxiety [52,53]	0.84 (525)	0.92 (482)
PHQ-2: Depression [54,55]	0.73 (529)	0.73 (490)
BRCS: Resiliency [56]	0.76 (541)	0.77 (507)
NGSES-8: Self-efficacy [57]	0.91 (524)	0.93 (491)

**Table 2:** Demographics and professional characteristics.

	Total, n = 535	Mean	SD
Age (years)		56	9.8
	Category	n	%
Sex	Male	289	52.4
	Female	262	47.5
Race/ethnicity ^a^	Asian or Pacific Islander	55	9.9
	Black or African American	17	3.1
	Hispanic or Latino	22	4.0
	White or Caucasian	441	79.2
	Other	17	3.1
Time zone	Eastern	222	46.0
	Central	111	23.0
	Mountain	43	8.9
	Pacific	104	1.0
Area	Urban	421	87.2
	Rural	62	12.8
Hotspot county	Yes	354	63.6
	No	203	36.5
Profession	Physician	473	85.4
	Nurse	71	12.8
	Psychologist	6	1.1
	Other	4	0.7
Setting ^b^	Acute care hospital	247	45.8
	Outpatient clinic/medical office	217	40.3
	Other ^c^	74	13.8
Specialty ^b^	Emergency medicine	55	10.7
	Internal medicine	111	21.6
	Pediatrics	108	21.0
	Primary care	60	11.7
	Other	180	35.0
Roles	Management or executive leadership	216	39.2
	Not in management or leadership	335	60.8
Length of practice ^b^	1–9 years	35	6.3
	10–21 years	190	34.3
	22–30 years	173	31.2
	≥31 years	156	28.2

^a^ Categories are not mutually exclusive; ^b^ Type of healthcare facility and physician specialties; ^c^ “Other” healthcare settings included academic institutions, ambulatory surgical centers, birthing centers, government offices, home health agencies, hospices, nursing homes, behavioral health centers, orthopedic/rehabilitation centers, correctional facilities, skilled nursing facilities, specialized outpatient services, diagnostic imaging/laboratory centers, and telehealth services.

**Table 3:** Care of COVID-19 patients and impact of COVID-19 on healthcare practice.

	Category	n	%
Direct COVID-19 patient care ^a^	Yes	393	71.2
	No	159	28.8
Impact of COVID-19 on practice ^b^	No impact	3	0.54
	Low impact	34	6.17
	Moderate impact	181	32.9
	High impact	241	43.7
	Severe impact	92	16.7
Percentage of patients seen daily with COVID-19 signs and symptoms ^c^	0%	100	18.1
	1–25%	384	69.6
	26–50%	48	8.7
	51–75%	16	2.9
	76–100%	4	0.7

^a^ Are you directly taking care of COVID-19 patients, including those who may be potentially infected with COVID-19? ^b^ Overall, how would you say COVID-19 has impacted your practice? ^c^ What percentage of patients that you see daily report having COVID-19-related signs and symptoms?

**Table 4:** Changes in self-reported psychosocial conditions, pre-COVID-19 and during COVID-19.

	Index Scores						T-Test ^a^					Correlation ^b^
Psychosocial Scales	Pre-COVID-19			During COVID-19								
	n	Mean	SD	n	Mean	SD	D Mean	D SD	DF	*t* Stat	*r*	*p*-Value
Burnout (M2QSS)	503	3.11	2.60	503	4.83	3.46	1.72	2.52	502	15.32	0.688	<0.001
Anxiety (GAD-7)	473	2.94	2.96	473	7.04	5.63	4.10	4.67	472	19.10	0.559	<0.001
Depression (PHQ-2)	471	0.67	0.96	471	1.82	1.76	1.15	1.50	470	16.65	0.525	<0.001
Resilience (BRCS)	501	15.76	3.03	501	14.86	3.30	−0.90	2.32	500	−8.69	0.733	<0.001
Self-Efficacy (NGSES-8)	480	34.11	4.21	480	31.89	5.33	−2.22	4.43	479	−10.98	0.591	<0.001

^a^ Pooled T-test of mean change; ^b^ Pearson correlation for each scale index score pre-COVID-19 with during COVID-19; Prob > |r| under H0: Rho = 0.

**Table 5:** Variables associated with burnout pre-COVID-19 (multiple regression).

	b	SD	*t* Stat	*p*-Value	sr^2^ Unique
Age	−0.052	0.013	−3.930	0.000	0.035
Assets/goals ^a,b^	−0.789	0.265	−2.980	0.003	0.020
Resilience pre-COVID-19 (BRCS)	−0.126	0.043	−2.900	0.004	0.019
Stressor—resources lacking ^a,c^	0.754	0.292	2.580	0.010	0.015
Mental health ^a,d^	2.225	0.837	2.660	0.008	0.016
Intercept	8.233				

^a^ Dummy variables (dichotomous); ^b^ Assets/goals: What individual or internal assets help you cope with the stress of treating COVID-19 patients? Achieving most of the goals that I set for myself; ^c^ Stressor—resources lacking: What aspects of your work make you feel the most stressed while treating COVID-19 patients? Lack of medical staff, medical equipment, personal protective equipment (PPE), and resources needed to treat COVID-19 patients; ^d^ Mental health: See Table S1 under Supplementary Materials for the survey questions pertinent to mental health.

**Table 6:** Variables associated with burnout during COVID-19 (multiple regression).

	b	SD	*t* Stat	*p*-Value	sr^2^ Unique
Age	−0.058	0.017	−3.440	0.001	0.025
COVID-19 high impact ^a,b^	−0.960	0.338	−2.840	0.005	0.017
Assets/goals ^a,c^	−0.811	0.337	−2.400	0.017	0.012
Resilience pre-COVID-19 (BRCS)	−0.233	0.051	−4.590	<0.001	0.044
Stressor—exhaustion ^a,d^	1.467	0.359	4.090	<0.001	0.035
Stressor—resource lack ^a,e^	1.067	0.377	2.830	0.005	0.017
Intercept	10.725				

^a^ Dummy variables (dichotomous); ^b^ COVID-19 high impact; ^c^ Assets/goals: What individual or internal assets help you cope with the stress of treating COVID-19 patients? Achieving most of the goals that I set for myself; ^d^ Stressor—exhaustion: What aspects of your work make you feel the most stressed while treating COVID-19 patients? Being exhausted from working long hours; ^e^ Stressor—resource lack: What aspects of your work make you feel the most stressed while treating COVID-19 patients? Lack of medical staff, medical equipment, personal protective equipment (PPE), and resources needed to treat COVID-19 patients.

**Table 7:** Percentage of perceived stressful aspects of treating COVID-19 patients, Sept.–Dec. 2020.

	Themes	n	%
Work aspects and stress ^a,b^	Experiencing uncertainty over the indefinite duration of the pandemic/uncertain return to normal	305	54.8
	Being anxious/fearful of infecting my family (exposing child, spouse, or older family member)	269	48.3
	Balancing my duty to my patients and my personal safety (fear of becoming infected)	248	44.3
	Being exhausted from working long hours	170	30.5
	Spending long hours in protective clothing	161	28.9
	Being frustrated by not being able to do much because of the lack of specific treatment for COVID-19	156	28.0
	Lack of medical staff, medical equipment, PPE, and resources needed to treat COVID-19 patients	135	24.2
	Other	109	19.6
	Learning about new COVID-19 cases on the news	99	17.8
	Witnessing my colleagues become infected with COVID-19	90	16.2
	Witnessing or learning of the death of my patients due to COVID-19	66	11.9

^a^ Categories are not mutually exclusive; ^b^ What aspects of your work make you feel the most stressed while treating COVID-19 patients?

**Table 8:** Percentage of perceived internal and external resilience assets and resources for coping with stress in treating COVID-19 patients, Sept.–Dec. 2020.

	Themes	n	%
Internal assets ^a,b^	Capacity or personal bandwidth to perform well during tough times	413	74.2
	Confidence in performing effectively on many different tasks	311	55.8
	Capacity to face and accomplish difficult tasks	291	52.2
	Obtaining outcomes that are important to me	260	46.7
	Successfully overcoming many challenges	254	45.6
	Achieving most of the goals that I set for myself	229	41.1
	Doing most tasks well compared with other people	189	33.9
	Succeeding at most any endeavor to which I set my mind	187	33.6
External resources ^a,c^	Emotional support from family, friends, and community	374	67.2
	Other	475	85.3
	Flexible work schedule/opportunities for time off work	350	62.8
	Positive attitude among my colleagues/peers	285	51.2
	Accurate guidance on infection prevention and control in my workplace	230	41.3
	Avoidance of news media about COVID-19	174	31.2
	Validation/praise from my supervisor or boss	117	21.0
	Positive social media messages from the community	84	15.1
	Availability of a place to quarantine without infecting family	65	11.7
	Access to a break room stocked with food	57	10.2
	Mental health support and resources in the workplace	55	9.9

^a^ Categories are not mutually exclusive; ^b^ What individual or internal assets help you cope with the stress of treating COVID-19 patients? ^c^ What external factors and resources listed below help you cope with the stress of treating COVID-19 patients?

## Data Availability

Restrictions apply to the availability of these data. Data were obtained from Reaction Data and are available from M.L.B.N. with the permission of Reaction Data.

## Supplementary Materials

The following supporting information can be downloaded at: https://www.mdpi.com/article/10.3390/ijerph20054414/s1: Table S1: Survey instrument; Table S2: Psychosocial impact of COVID-19 in epidemiological COVID-19 hotspots; Table S3: Reporting of psychosocial conditions as to gender; Table S4: Variables associated with resilience during COVID-19 (multiple regression).
