# Supplementary material for: COVID-19 and Psychosocial Well-Being: Did COVID-19 Worsen U.S. Frontline Healthcare Workers’ Burnout, Anxiety, and Depression?
Source: Int J Environ Res Public Health. 2023 Mar 1;20(5):4414. doi: 10.3390/ijerph20054414 (PMC10002248; doi:10.3390/ijerph20054414)
Supplement: Supplementary file 1 [file ijerph-20-04414-s001.zip › ijerph-2207348-supplementary.pdf]

COVID-19 and Psychosocial Well-Being: Did COVID-19 Worsen U.S. Frontline Healthcare Workers' Burnout, Anxiety, and Depression?

M. Lelinneth B. Novilla [1,*], Victor B. A. Moxley [2,*], Carl L. Hanson [1], Alisha H. Redelfs [1,*], Jeffrey Glenn [1], Paola G. Donoso Naranjo [1], Jenna M.S. Smith [1], Lynneth Kirsten B. Novilla [1], Sarah Stone [1] and Rachel Lafitaga [1]

Supplementary Materials

Table S1. Survey Instrument

Question	Response Options
1. *What is your profession?	
	a. Medical Doctor (MD)<br>b. Doctor of Osteopathy (DO)<br>c. Nurse Practitioner (NP)<br>d. Physician Assistant (PA)<br>e. Registered Nurse (RN)<br>f. Medical Resident/Intern<br>g. Other, please specify: _________
2. *What is your medical specialty or subspecialty?	a. Emergency Medicine<br>b. Family Practice<br>c. Pediatrics<br>d. Internal Medicine<br>e. Pulmonology<br>f. Neurology<br>g. Gastroentorology<br>h. Infectious Disease<br>i. Nephrology<br>j. Endocrinology<br>k. Oncology<br>l. Psychiatry<br>m. Other, please specify: __________
3. *What is your current job level or employment rank? *Please click the box that most accurately describes your job level.*	A. Executive Management<br>a. Senior Executive<br>b. Executive<br>B. Middle Management<br>a. Senior Director<br>b. Director<br>C. Managers & Advisors<br>a. Senior Manager/Advisor<br>b. Manager/Advisor<br>D. Medical Staff<br>a. Senior<br>b. Associate<br>c. Intermediate<br>d. Entry-level
4. **Health care setting includes various locations that offer health care services.* What type	a. Acute Care Hospital<br>b. Urgent Care Center<br>c. Outpatient Clinic/Medical Office<br>d. Telehealth

of health care setting do you work in?	e. Orthopedic and Other Rehabilitation Center<br>f. Imaging and Radiology Center<br>g. Specialized Outpatient Service (hemodialysis, podiatry, chemotherapy, endoscopy, pain management clinic)<br>h. Mental Health & Addiction Treatment Center<br>i. Nursing Home<br>j. Skilled Nursing Facility<br>k. Dental Office<br>l. Diabetes Education Center<br>m. Birthing Center<br>n. Hospice Home<br>o. Other, please specify: _______
5. *What is the ZIP code of your medical facility?	__________
6. *What is your gender?	a. Male<br>b. Female<br>c. Other, please specify: __________
7. *What is your year of birth?	______
8. *How long have you been practicing in your current profession or medical specialty?	a. 1-3 years<br>b. 4-6 years<br>c. 7-9 years<br>d. 10-12-years<br>e. 13-15 years<br>f. 16-18 years<br>g. 19-21 years<br>h. 22-24 years<br>i. 25-27 years<br>j. 28-30 years<br>k. 31 or more years
9. *Which of the following best describes your race/ethnicity? *Select all that apply.*	a. Asian or Pacific Islander<br>b. Black or African American<br>c. Hispanic or Latino<br>d. Native American or Alaskan Native<br>e. White or Caucasian<br>f. Other
10. Are you directly taking care of COVID-19 patients including those who may be potentially infected with COVID-19? *Directly taking care of COVID-19 patients or those who may be potentially infected include responsibilities related to testing, hospital/clinic triage, laboratory diagnosis, radiology and imaging,*	a. Yes<br>b. No (proceed to Q#____)

respiratory therapy, treatment, nursing, rehabilitation, consults or referrals.	
11. Overall, how would you say COVID-19 has impacted your practice?	a. No impact<br>b. Low impact<br>c. Moderate impact<br>d. High impact<br>e. Severe impact
12. What percentage of the patients that you see daily has complaints of COVID-19-related signs and symptoms?	a. 0%<br>b. 1-25%<br>c. 25-50%<br>d. 51-75%<br>e. 76-100%

Over the last 2 weeks, how often have you been bothered by any of the following problems?

13. Little interest or pleasure in doing things[PHQ-2;1,2]

	0-Not at All	1-Several Days	2-More than Half the Days	3-Nearly Every Day
Now	○	○	○	○
Before COVID-19	○	○	○	○

14. Feeling down, depressed, or hopeless[PHQ-2;1,2]

	0-Not at All	1-Several Days	2-More than Half the Days	3-Nearly Every Day
Now	○	○	○	○
Before COVID-19	○	○	○	○

15. Feeling nervous, anxious, or on edge[GAD-7;3,4]

	0-Not at All	1-Several Days	2-More than Half the Days	3-Nearly Every Day
Now	○	○	○	○
Before COVID-19	○	○	○	○

16. Not being able to stop or control worrying[GAD-7;3,4]

	0-Not at All	1-Several Days	2-More than Half the Days	3-Nearly Every Day
Now	○	○	○	○
Before COVID-19	○	○	○	○

17. Worrying too much about different things[GAD-7;3,4]

	0-Not at All	1-Several Days	2-More than Half the Days	3-Nearly Every Day
Now	○	○	○	○
Before COVID-19	○	○	○	○

18. Trouble relaxing[GAD-7;3,4]

	0-Not at All	1-Several Days	2-More than Half the Days	3-Nearly Every Day
Now	○	○	○	○
Before COVID-19	○	○	○	○

19. Being so restless that it's hard to sit still[GAD-7;3,4]

	0-Not at All	1-Several Days	2-More than Half the Days	3-Nearly Every Day
Now	○	○	○	○
Before COVID-19	○	○	○	○

20. Becoming easily annoyed or irritable[GAD-7;3,4]

	0-Not at All	1-Several Days	2-More than Half the Days	3-Nearly Every Day
Now	○	○	○	○
Before COVID-19	○	○	○	○

21. Feeling afraid as if something awful might happen[GAD-7;3,4]

	0-Not at All	1-Several Days	2-More than Half the Days	3-Nearly Every Day
Now	○	○	○	○
Before COVID-19	○	○	○	○

Please indicate how often you have felt the following:

22. "I feel burned out from my work."[M2QSS,5]

	0 - Never	1 - A few times a year or less	2 - Once a month or less	3 - A few times a month	4 - Once a week	5 - A few times a week	6 - Every day
Now	○	○	○	○	○	○	○
Before COVID-19	○	○	○	○	○	○	○

23. "I have become more callous toward people since I took this job." [M2QSS,5]

	0 - Never	1 - A few times a year or less	2 - Once a month or less	3 - A few times a month	4 - Once a week	5 - A few times a week	6 - Every day
Now	○	○	○	○	○	○	○
Before COVID-19	○	○	○	○	○	○	○

24. What aspects of your work make you feel the most stressed while treating COVID-19 patients? *Select all that apply.*

a. Witnessing my colleagues become infected with COVID-19
b. Being fearful of infecting my family
c. Balancing my duty to my patients and my personal safety (fear of becoming infected)
d. Witnessing or learning of the death of my patients due to COVID-19
e. Experiencing uncertainty of when the outbreak will be contained
f. Learning of/not being able to do much because of the lack of specific treatment for COVID-19
g. Learning about new COVID-19 cases on the news
h. Being exhausted from working long hours
i. Spending long hours in protective clothing
j. Lacking in medical staff, medical equipment, personal protective equipment, and resources needed to treat COVID-19 patients
k. Other, please specify: ________

Consider how well the following statements describe your behavior and actions on a scale from 1 to 5, where 1 means the statement does not describe you at all and 5 means it describes you very well.

25. I look for creative ways to alter difficult situations[BRCS-4,58]

	1 - Does not describe me at all	2	3	4	5 - Describes me very well
Now	○	○	○	○	○
Before COVID-19	○	○	○	○	○

26. Regardless of what happens to me, I believe I can control my reaction to it[BRCS-4;6]

	1 - Does not describe me at all	2	3	4	5 - Describes me very well
Now	○	○	○	○	○
Before COVID-19	○	○	○	○	○

27. I believe I can grow in positive ways by dealing with difficult situations [BRCS-4;6]

	1 - Does not describe me at all	2	3	4	5 - Describes me very well
Now	○	○	○	○	○
Before COVID-19	○	○	○	○	○

28. I actively look for ways to replace the losses I encounter in life [BRCS-4;6]

	1 - Does not describe me at all	2	3	4	5 - Describes me very well
Now	○	○	○	○	○
Before COVID-19	○	○	○	○	○

29. What external factors and resources listed below help you cope with the stress of treating COVID-19 patients? *Select all that apply.*

a. Opportunities for time off from work
b. Access to a break room stocked with food
c. Availability of place to quarantine without infecting family
d. Mental health support and resources in workplace
e. Emotional support from family, friends or community members
f. Positive social media messaging from the community
g. Positive attitudes among my colleagues
h. Validation/praise from my supervisor or boss
i. Accurate guidance on infection prevention and infection control in my workplace
j. Avoidance of news media about COVID-19

	k. Other, please specify: ________
30. What individual or internal assets help you cope with the stress of from treating COVID-19 patients? *Select all that apply.*	a. Achieving most of the goals that I set for myself.<br>b. Facing and accomplishing difficult tasks<br>c. Obtaining outcomes that are important to me.<br>d. Succeeding at most any endeavor to which I set my mind.<br>e. Successfully overcoming many challenges.<br>f. Confidence in performing effectively on many different tasks.<br>g. Doing most tasks well compared to other people.<br>h. Performing well even when things are tough.

Using a 5-point rating scale (1 = strongly disagree; 2 = disagree; 3 = neither agree nor disagree; 4 = agree; 5 = strongly agree), rate how much you agree with each of the statements listed below:

31. "I will be able to achieve most of the goals that I set for myself."[NGSES-8;7]

	1-Strongly Agree	2-Disagree	3-Neither Agree nor Disagree	4-Agree	5-Strongly Agree
Now	○	○	○	○	○
Before COVID-19	○	○	○	○	○

32. "When facing difficult tasks, I am certain that I will accomplish them."[NGSES-8;7]

	1-Strongly Agree	2-Disagree	3-Neither Agree nor Disagree	4-Agree	5-Strongly Agree
Now	○	○	○	○	○
Before COVID-19	○	○	○	○	○

33. "In general, I think that I can obtain outcomes that are important to me."[NGSES-8;7]

	1-Strongly Agree	2-Disagree	3-Neither Agree nor Disagree	4-Agree	5-Strongly Agree
Now	○	○	○	○	○
Before COVID-19	○	○	○	○	○

34. "I believe I can succeed at most any endeavor to which I set my mind."[NGSES-8;7]

	1-Strongly Agree	2-Disagree	3-Neither Agree nor Disagree	4-Agree	5-Strongly Agree
Now	○	○	○	○	○

Before COVID-19	○	○	○	○	○

35. "I will be able to successfully overcome many challenges."[NGSES-8;7]

	1-Strongly Agree	2-Disagree	3-Neither Agree nor Disagree	4-Agree	5-Strongly Agree
Now	○	○	○	○	○
Before COVID-19	○	○	○	○	○

36. "I am confident that I can perform effectively on many different tasks."[NGSES-8;7]

	1-Strongly Agree	2-Disagree	3-Neither Agree nor Disagree	4-Agree	5-Strongly Agree
Now	○	○	○	○	○
Before COVID-19	○	○	○	○	○

37. "Compared to other people, I can do most tasks very well."[NGSES-8;7]

	1-Strongly Agree	2-Disagree	3-Neither Agree nor Disagree	4-Agree	5-Strongly Agree
Now	○	○	○	○	○
Before COVID-19	○	○	○	○	○

38. "Even when things are tough, I can perform quite well."[NGSES-8;7]

	1-Strongly Agree	2-Disagree	3-Neither Agree nor Disagree	4-Agree	5-Strongly Agree
Now	○	○	○	○	○
Before COVID-19	○	○	○	○	○

References:

1. Li, M.M.; Friedman, B.; Conwell, Y.; Fiscella, K. *Validity of the Patient Health Questionnaire 2 (PHQ-2) in Identifying Major Depression in Older People* . *J Am Geriatr Soc.* 2007; 55:596-602.
2. Advancing Integrated Mental Health Solutions Center (AIMS). University of Washington Psychiatry and Behavioral Sciences Division of Population Health. Patient Health Questionnaire 2 (PHQ-2). Available at:

https://aims.uw.edu/resource-library/patient-health-questionnaire-2-phq-2. Updated 2021 (accessed July 23, 2020).
3. Ruiz, M.A.; Zamorano, E.; García-Campayo, J.; Pardo, A., Freire, O.; Rejas, J. Validity of the GAD-7 scale as an outcome measure of disability in patients with generalized anxiety disorders in primary care. *J Affect Disord.* 2011;128(3): 277–286. doi: 10.1016/j.jad.2010.07.010.
4. Spitzer, R.L.; Kroenke, K.; Williams, J.B.W.; Löwe, B. A brief measure for assessing Generalized anxiety disorder: the GAD-7. *Arch Intern Med.* 2006;166(10):1092–1097. doi:10.1001/archinte.166.10.1092.
5. Li-Sauerwine, S.; Rebillot, K.; Melamed, M.; Addo, N.; Lin, M. A 2-question summative score correlates with the Maslach Burnout Inventory. *West. J. Emerg. Med.* 2020; 21(3): 610-617. doi: 10.5811/westjem.2020.2.4513. Accessed May 22, 2020. http://escholarship.org/uc/uciem_westjem.
6. Sinclair, V. G.; Wallston, K. A. (2004). The Development and Psychometric Evaluation of the Brief Resilient Coping Scale. Assessment, 11(1), 94–101. https://doi.org/10.1177/1073191103258144.
7. Chen, G.; Gully, S.M.; Eden, D. Validation of a new general self-efficacy scale. *Organ Res Methods*. 2001; *4*(1): 62-83. Available at: https://sparqtools.org/mobility-measure/new-general-self-efficacy-scale/#all-survey-questions (accessed July 23, 2020).

Table S2. Psychosocial impact of COVID-19 in epidemiological COVID-19 hotspots.

	Difference in Index Scores Female to Male t-Test[a]			
Psychosocial Scales	Pre-COVID-19		During COVID-19	
	Difference	*P- value*	*Difference*	*P- value*
Burnout (M2QSS)	-0.406	.079	0.290	.359
Anxiety (GAD-7)	-0.478	.078	-0.010	.985
Depression (PHQ-2)	-0.084	.345	-0.017	.916
Resilience (BRCS)	0.110	.654	0.343	.260
Self-Efficacy (NSES-8)	0.241	.524	0.506	.306

[a] Pooled t-test of mean change

Table S3. Reporting of psychosocial conditions as to gender.

	Difference in Index Scores Female to Male t-Test[a]			
Psychosocial Scales	Pre-COVID-19		During COVID-19	
	Difference	*P- value*	*Difference*	*P- value*
Burnout (M2QSS)	0.359	.109	1.27	<.001
Anxiety (GAD-7)	0.642	.014	2.98	<.001
Depression (PHQ-2)	0.098	.251	0.557	<.001
Resilience (BRCS)	0.232	.375	0.689	.022
Self-Efficacy (NSES-8)	0.195	.600	0.910	.059

[a] Pooled t-test of mean change

Table S4. Variables associated with resilience during COVID (Multiple Regression)*.

	Coefficient	*P-value*
Age	-0.052	.000
Goals[a]	1.409	<.001
Difficult tasks[b]	-0.126	.004
Outcomes[c]	-0.820	.019
Overcoming[d]	1.031	.003
Confidence[e]	0.861	.014
Uncertainty[f]	-0.904	.005
Resources lacking[g]	-1.004	.006
Intercept	13.891	

*Overall model significance $p < .001$

[a] Individual/Internal Assets: "Achieving most of the goals that I set for myself"

[b] Individual/Internal Assets: "Facing and accomplishing difficult tasks"

[c] Individual/Internal Assets: "Obtaining outcomes that are important to me"

[d] Individual/Internal Assets: "Successfully overcoming many challenges"

[e] Individual/Internal Assets: "Confidence in performing effectively on many different tasks"

[f] Work Stress: "Experiencing uncertainty of when the outbreak will be contained"

[g] Work Stress: "Lacking in medical staff, medical equipment, personal protective equipment, and resources needed to treat COVID-19 patients"
